# Use of comparative effectiveness research for similar Chinese patent medicine for angina pectoris of coronary heart disease: a new approach based on patient-important outcomes

**DOI:** 10.1186/1745-6215-15-84

**Published:** 2014-03-19

**Authors:** Hongbo Cao, Jingbo Zhai, Wei Mu, Xiang Lei, Hongxia Cao, Chunxiang Liu, Hongcai Shang

**Affiliations:** 1 88, Yuquan road, Tianjin Institute of Clinical Evaluation, Tianjin University of Traditional Chinese Medicine, Tianjin 300193, China; 2 312, Anshanxidao road,Center for Evidence-Based Medicine, Tianjin University of Traditional Chinese Medicine, Tianjin 300193, China; 3 518, Anxi roadGansu Hospital of Traditional Chinese Medicine, Lanzhou 730050, China

**Keywords:** Angina pectoris, Chinese patent medicine, Comparative effectiveness research, Patient-important outcomes

## Abstract

**Background:**

The practice of traditional Chinese medicine (TCM) has a profound history in many Asian countries. TCM syndrome is a set of characteristic physical signs and symptoms shared by a group of patients. Syndrome diagnosis and treatment assignment according to the identified TCM syndrome is a long-held practice of Chinese medicine. Owing to its distinctive way of interpreting illness and administering care, medical practitioners not well educated in TCM theories and practices are generally incapable of giving out prescriptions for Chinese patent drugs. Currently, the existence of a multitude of Chinese patent drugs marked with largely identical indications is further complicating this situation.

**Methods:**

In this multicenter, randomized, controlled, double-blind, double-dummy clinical trial, in which we will use the comparative effectiveness research method, we will compare the efficacy of two commonly used Chinese patent medicines for angina patients diagnosed with qi deficiency and blood stasis syndrome. A total of 160 patients will be recruited and randomly assigned to receive either (1) QiShenYiQi dripping pills, Tongxinluo placebo and routine medication or (2) Tongxinluo capsules, QiShenYiQi placebo and routine medication. These treatment regimens will be carried out for 4 weeks, followed by a 10-day washout period and a 4-week crossover phase in which the treatments in the two patient groups will be exchanged. Patients will be allowed to choose symptoms that matter most to them and will be grouped accordingly. Patient-reported outcomes such as the Seattle Angina Questionnaire score and the 15-point Likert scale score will be measured and reported. The minimally clinical important difference will be calculated and used for efficacy assessment, and correspondence analysis will be performed to identify the best indications for each drug.

**Discussion:**

The goal of the study is to establish a methodology for the precise identification of the characteristic indications for which a Chinese patent drug is most effective. The findings of this study will inform the practicality of the proposed evaluation method.

**Trial registration:**

Chinese clinical trials register Chi CTRTTRCC13003732

## Background

### Traditional Chinese medicine interpretation of angina and therapeutic rules

Syndrome differentiation, also termed *pattern diagnosis* (*Bian Zheng* in Chinese pinyin), is one of the kernel theories of traditional Chinese medicine (TCM). The TCM syndrome, or *Zheng*, is a characteristic profile of clinical signs and symptoms manifested by a group of patients. The clinical efficacy of Chinese medicine relies heavily on the correct diagnosis of a specific TCM syndrome. According to the 2002 Guidelines for Clinical Research of Chinese Medicine (new drug) [1] released by the Chinese Food and Drug Administration patients with angina pectoris can be diagnosed on the basis of any one of the following eight TCM patterns: (1) qi deficiency and blood stasis syndrome, (2) heart blood stasis obstruction syndrome, (3) qi stagnation and blood stasis syndrome, (4) syndrome of phlegm obstruction in the heart vessel, (5) syndrome of congealing yin cold, (6) dual-deficiency of qi and yin syndrome, (7) heart and kidney yin deficiency syndrome and (8) yang qi debilitation syndrome. Among these criteria, the qi deficiency and blood stasis syndrome is most typically observed in Chinese angina patients.

For the management of angina pectoris patients with qi deficiency and blood stasis syndrome, herbal remedies intended to invigorate qi, activate blood circulation and transform stasis will be administered. The main function of the herbal remedy is to improve clinical signs and symptoms of the patients.

### Comparative effectiveness research and traditional Chinese medicine

The concept of comparative effectiveness research (CER) was initiated in the 1990s by Mark Boutin, the deputy executive president and chief operating officer of the US National Health Council [2]. In the years that followed, CER has been used to aid public health policy-making and introduced into the field of clinical research in a number of countries [3]. In May 2011, Claudia M Witt (Institute of Social Medicine, Epidemiology and Health Economics, Charité University Medical Center, Berlin, Germany) proposed introducing CER techniques into TCM research at the sixth annual meeting of the International Society of Complementary Medicine Research [4].

### Efficacy evaluation based on patient-reported outcomes

A patient-reported outcome (PRO) is a report directly from the patient, without influences from doctors or others, about how they function or feel in relation to their health status and the treatment they are receiving [5]. The PRO measurement tools are developed to provide insights from the patient’s perspective regarding the impact of therapeutic interventions on their health. Patient-important outcomes (PIOs) are a selection of outcome measures that are considered most relevant to the patients. Examples of PIOs include pain, fatigue, quality of life and death. Efforts to assess PIOs for a particular disease or condition and to promote the use of PIOs in clinical research settings facilitate effective interpretation of the results by emphasizing the evaluation of treatment effectiveness. The integration of PIOs and PROs into Comparative Effectiveness Research could be a useful tool for the assessment of outcomes requiring patients’ subjective perceptions and judgments, such as consumer satisfaction and health-related quality of life [6].

### Minimal clinically important differences and patient-reported outcome measurements

Minimal clinically important differences (MCIDs) are self-perceived scores that signify clinically significant improvements in PROs that can be used to identify the minimal changes in a specific aspect of a patient’s health [7], and it is believed to reflect the smallest change in the PRO evaluations of a clinical intervention that is meaningful to the patient.

Distribution-based methods for the determination of MCIDs have been widely used and can be subdivided into the effect size (ES) and the standard error of measurement (SEM). The latter measurement is particularly recommended for its relative independence from sample source and stability across different studies [8,9]. In our present study, changes in PROs will be calculated using the SEM, which will represent patients’ self-reported improvements in symptoms or health-related function.

### Traditional Chinese medicine for angina pectoris due to coronary heart disease

Angina pectoris is one of the most common symptoms of coronary heart disease (CHD) and afflicts a large portion of CHD patients. It has been found over the course of years of clinical practice that TCM is effective in treating angina. Because the qi deficiency and blood stasis syndrome together have been identified as a major TCM syndrome among patients with angina, in this CER study we will select two Chinese patent drugs most frequently used for treating angina patients diagnosed with this common syndrome. The patent drugs to be compared are QiShenYiQi (QSYQ) dripping pills (Tasly Pharmaceutical Co Ltd, Tianjin, China) and Tongxinluo (TXL) capsules (Yiling Pharmaceutical Co Ltd, Shijiazhuang City, China). QSYQ pills are composed mainly of *Radix Astragali*, *Radix Salvia miltiorrhiza*, *Radix Notoginseng* and *Lignum Dalbergia Odorifera*. TXL capsules are an herbal mixture of *Radix Ginseng*, Hirudo, Quan Xie *Eupolyphaga seu Steleophaga*, *Scolopendra*, Periostracum Cicadae, Radix Paeoniae rubra, Lignum Santali Albi, *Lignum Dalbergia Odorifera*, frankincense, *Semen Ziziphi spinosae* and borneol.

## Objectives

We are conducting a multicenter, randomized, controlled, double-blind, double-dummy clinical trial to compare the effectiveness of two Chinese patent medicines for angina patients diagnosed with qi deficiency and blood stasis syndrome. The ultimate goal is to test the proposed methodology for the individualized evaluation of the therapeutic effects of Chinese patent drugs and for distinguishing between drugs targeting a multitude of similar indications.

To fulfill these objectives in our present study, we highlight the introduction of trial participants’ therapeutic needs and willingness in terms of group allocation and the use of CER methodology. Specifically before being entered into the randomization process, all eligible participants with qi deficiency and blood stasis syndrome will first be allowed to choose from among four prespecified groups of symptoms which they would most like to be addressed. The outcomes for these symptoms are considered PIOs and will be assessed using PRO instruments. Furthermore, we will test the use of the CER method to compare the curative effects of QSYQ dripping pills and TXL capsules, two widely used Chinese patent medicines for angina patients with qi deficiency and blood stasis syndrome. By analyzing and comparing the effects of the two drugs for each symptom group, we aim to summarize the characteristics of the drugs’ curative effects and identify the best targeted indications for each drug.

## Methods

### Study design

**Figure 1:**
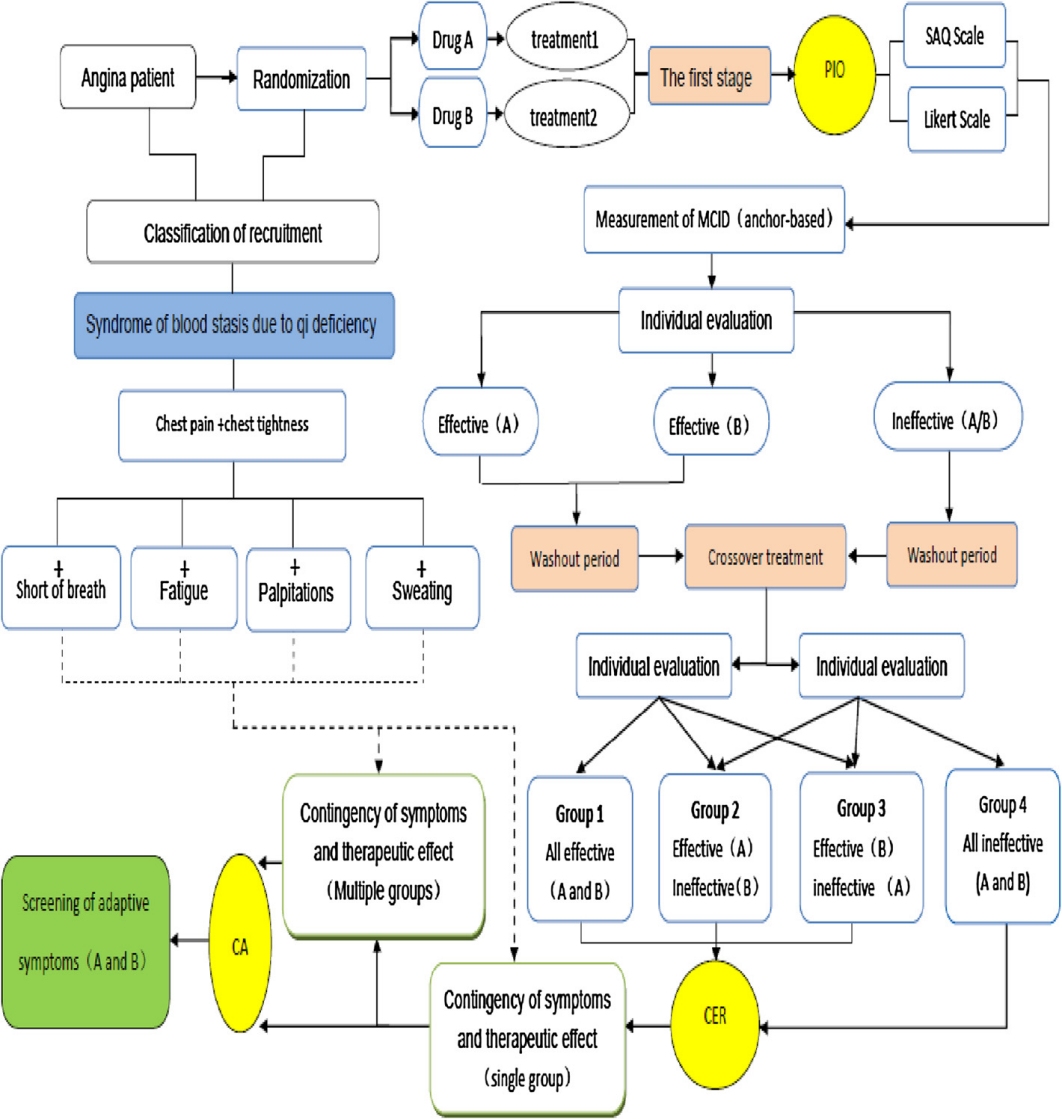
**Study flowchart.** CA, Correspondence analysis; CER, Comparative effectiveness research; MCID, Minimal clinically important difference; PIO, Patient-important outcome; SAQ, Seattle Angina Questionnaire.

We will conduct a multicenter, randomized, crossover, double-blind, double-dummy clinical trial. A flowchart of the study protocol is shown in Figure 1.

## Ethical approval

This study has been approved by the medical ethics committee of Tianjin University of Traditional Chinese Medicine (registration number TJUTCM-EC20130004).

### Setting

Participants will be recruited simultaneously at the cardiology departments of four hospitals in Tianjin City in mainland China.

### Participant recruitment

Potentially eligible angina patients will be provided with general information regarding the trial, the objectives of the research and the rights and obligations of the participants at the first visit. Those who are willing to participate will be asked to give their informed consent to participate before being entered into the screening process. Participants will then be included or excluded according to the predefined inclusion and exclusion criteria.

### Diagnostic criteria for traditional Chinese medicine patterns

According to the 2002 Guidelines for Clinical Research of Chinese Medicine (new drug), any patient presenting with the primary symptoms mentioned below and at least one of the secondary symptoms, with typical tongue and pulse presentations, will be diagnosed with qi deficiency and blood stasis syndrome. Primary symptoms include chest pain and chest tightness. Secondary symptoms include shortness of breath, fatigue, palpitations and/or spontaneous perspiration. .

#### *Inclusion criteria*

1. Patients ages 40 to 75 years old

2. Provision of signed informed consent

3. Diagnosis of angina pectoris due to CHD (stable angina, New York Heart Association (NYHA) classes I to III)

4. TCM pattern diagnosis of qi deficiency and blood stasis

#### *Exclusion criteria*

1. Patients younger than 40 or older than 75 years old

2. Diagnosis of NYHA class IV CHD

3. Uncontrolled NYHA class III hypertension (systolic blood pressure ≥180 mmHg and/or diastolic blood pressure ≥110 mmHg)

4. Presence of severe arrhythmia

5. Patients with serious primary hepatic, renal, hematologic or mental illness and those with malignant tumors

6. Presence of active peptic ulcers and other hemorrhagic diseases

7. Pregnancy or lactation

8. Patients with other complications who should not be included in the trial as adjudged by the recruiting personnel

9. Patients with an allergic constitution or a history of allergy to the investigational drug

10. Participation in another clinical trial, either currently or within the past 3 months

#### *Exclusion criteria*

1. Incidence of serious adverse events.

2. Major design flaws or serious deviation from research protocol

3. Cancellation of the study by administrative authorities

### Patient grouping and treatment allocation

Using stratified and blocked randomization, we will first categorize eligible participants into four groups on the basis of their varied choice of secondary symptoms to be addressed, and then we will randomly assign them to one of the two treatment arms at a ratio of 1:1. To minimize imbalances across groups, gender will also be used as a stratification factor. The random number sequence will be generated by a third-party statistician using SAS version 9.1 software (SAS Institute, Cary, NC, USA).

The four symptom combination groups, each representing the signs a patient most wishes to bring under control, were formed according to the characteristic physical manifestations of the qi deficiency and blood stasis syndrome defined in the 2002 Guidelines for Clinical Research of Chinese Medicine (new drug). The grouping criteria for patients with varying therapeutic needs resulted in the following four symptom combination groups:

1. Combination 1: chest pain + chest tightness + shortness of breath

2. Combination 2: chest pain + chest tightness + fatigue

3. Combination 3: chest pain + chest tightness + palpitations

4. Combination 4: chest pain + chest tightness + spontaneous perspiration

### Sample size calculation

Sample size estimation is based on the results of a pilot study that showed the difference in changes in Seattle Angina Questionnaire (SAQ) scores of 2 ± 2.5 between the group receiving QSYQ dripping pills and the group taking TXL capsules. Assuming a two-sided *P*-value of 0.05, 90% power and calculating for a dropout rate of 20%, one would need to have approximately 160 patients with 80 in each treatment group. We used the PAST software program to calculate these statistics (http://folk.uio.no/ohammer/past/) [10]. Therefore, we plan to recruit 40 patients for each of the four symptom combinations, from among whom 20 will be assigned to treatment arm A (QSYQ) and 20 to treatment arm B (TXL). In all, 160 patients will be recruited.

### Randomization

Randomization of the trial participants will be completed using an independent data center using an interactive voice response system.

#### *Blinding and allocation concealment*

The test drug will be coded and placed in indistinguishable containers by specially assigned personnel who will not participate in other procedures in the trial. Drug assignments will be enclosed in sealed, opaque envelopes and kept confidential by the trial management board. Thus, the patients, clinicians, participating nurses, trial coordinators, outcome assessors and statisticians will be blinded to treatment assignment.

#### *Baseline assessment*

General information about the patient’s gender, age and heart function will be collected and assessed after eligibility screening to ensure balanced baseline values.

#### *Assignment of interventions*

In this study, we adopt a crossover design which involves three key phases: a 4-week treatment period, a 10-day washout period and the crossover of treatment for another 4 weeks:

1. The first treatment period (4 weeks): A total of 160 participants will be randomly assigned to receive treatment A or treatment B for 4 weeks.

1.a. Treatment A: QSYQ dripping pills + TXL placebo + routine medication

1.b. Treatment B: TXL capsules + QSYQ placebo + routine medication

2. Washout phase (10 days): After the first round of treatment, patients in both groups will receive QSYQ placebo and TXL placebo plus routine medication for 10 days.

3. Crossover of treatments (4 weeks): In this stage, patients formerly assigned to treatment A will be switched to treatment B and those assigned to treatment B will be switched to treatment A.

#### *Routine medication*

Routine medications will include aspirin, angiotensin-converting enzyme inhibitors or angiotensin receptor blockers, β-receptor blockers, statins and nitrates.

#### *Concomitant medications*

1. Dihydropyridines and diuretics

2. Antidiabetic drugs

3. Digoxin and diuretics

### Measurement tools and time of data capture

The SAQ and 15-point Likert scale scores will be used to collect PROs immediately following the first treatment period and the crossover treatment period. The minimal clinically important differences (MCIDs) of the SAQ and the Likert scale scores at these two time points will be calculated for efficacy evaluation and comparison.

#### *Efficacy assessment tools*

**Figure 2:**
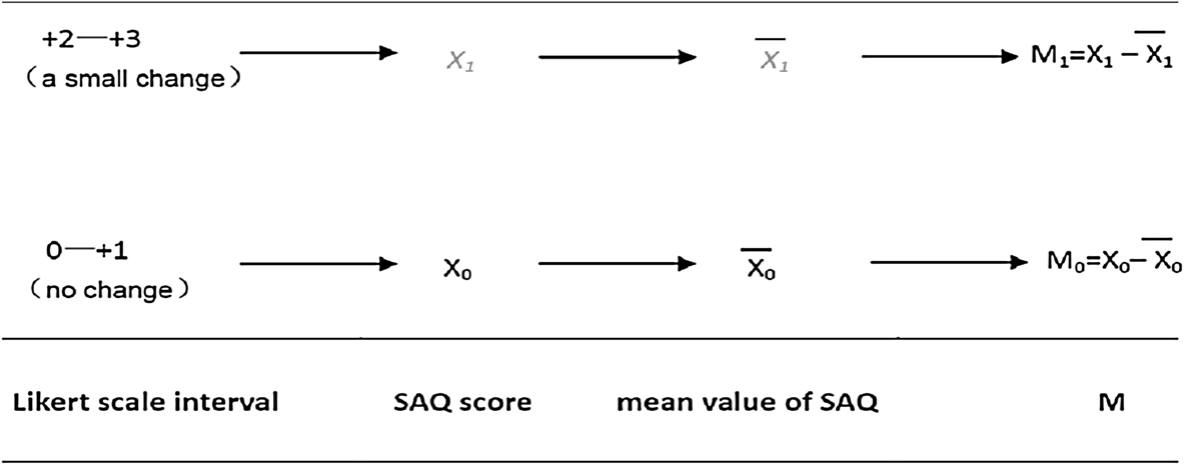
**Calculation of the M value.** SAQ, Seattle Assessment Questionnaire.

1. MCID calculation [11]: The MCID value will be calculated using the SEM method and following the steps outlined below:

1.a. Calculation of M (M_0_ and M_1_) (Figure 2):

1.a.i. Find patients receiving treatment A (or treatment B) whose self-reported Likert scores are within the range of +2 to +3.

1.a.ii. Collect the corresponding SAQ score of these patients, use X_1_ to refer to it and calculate the mean value $X{\left(—\right)}_{1}$.

1.a.iii. Find patients receiving Treatment A (or Treatment B) whose self-reported Likert scores are within the range of 0 to +1.

1.a.iv. Collect the SAQ scores of these patients, use X_0_ to refer to it and calculate the mean value X_0_

1.a.v. $M_{0}=X_{0}-X{\left(—\right)}_{0}$

1.a.vi. $M_{1}=X_{1}-X{\left(—\right)}_{1}$

2. Calculation of SEM (SEM_0_ and SEM_1_) [7,12,13].

$$\mathrm{SEMO}=\sqrt{\sum M0^{2}/\left(n-1\right)}\times \sqrt{1-r}$$

$$\mathrm{SEM}1=\sqrt{\sum M1^{2}/\left(n-1\right)}\times \sqrt{1-r}$$

n:sample size; r:the reliability coefficient of SAQ

3. Calculation of MCID We will calculate the MCIDs for treatment A (referred to as MCID1) and treatment B (referred to as MCID2) during the first treatment period and for treatment A (referred to as MCID3) and treatment B (referred to as MCID4) during the crossover period.

MCID, Minimal clinically important difference; SEM, Standard error of measurement.

$$\mathrm{MCID}\;=\;\sqrt{\mathrm{SEM}O^{2}+\mathrm{SEM}1^{2}}$$

4. Individualized efficacy evaluation upon the first round of treatment: Treatment interventions will be defined as either effective or ineffective by comparison of the patient’s personal SAQ score with the calculated MCID value.

4.a. Treatment A will be defined as effective if a patient receiving it has a SAQ score greater than the MCID1 score.

4.b. Treatment B will be defined as effective if a patient receiving it has a SAQ score greater than the MCID2 score.

4.c. A treatment will be adjudged ineffective if a patient receiving it reports a SAQ score lower than the corresponding MCID value (MCID1 or MCID2).

5. Individualized efficacy evaluation upon crossover of treatment: Because of the complexity of the study design, all the patients will be categorized into four groups by using the following assessment tools:

5.a. Group 1: Both treatments A and B are effective for patients in this group:

5.a.i. For patients who first receive treatment A and then treatment B, the first SAQ score > MCID1 and the second SAQ score > MCID4

5.a.ii. For patients who first receive treatment B and then treatment A, the first SAQ score > MCID2 and the second SAQ score > MCID3

5.b. Group 2: Treatment A is effective and treatment B is ineffective for patients in this group:

5.b.i. For patients who first receive treatment A and then treatment B, the first SAQ score > MCID1 and the second SAQ score < MCID4

5.b.ii. For patients who first receive treatment B and then treatment A, the first SAQ score > MCID2 and the second SAQ score < MCID3.

5.c. Group 3: Treatment B is effective and treatment A is ineffective for patients in this group:

5.c.i. For patients who first receive treatment A and then treatment B, the first SAQ score < MCID1 and the second SAQ score > MCID4

5.c.ii. For patients who first receive treatment B and then treatment A, the first SAQ score < MCID2 and the second SAQ score > MCID3

5.d. Group 4: Both treatments A and B are ineffective for patients in this group:

5.d.i. For patients who first receive treatment A and then treatment B, the first SAQ score < MCID1 and the second SAQ score < MCID4

5.d.ii. For patients who first receive treatment B and then treatment A, the first SAQ score < MCID2 and the second SAQ score < MCID3

### Statistical analysis

#### *Test for baseline balance*

Baseline data will be analyzed using an independent *t*-test, analysis of variance and the *χ*^2^ test to check whether the randomization has resulted in equal distributions of known confounding factors, such as age, gender, living status and education level.

#### *Comparison of the curative effects of treatment A and treatment B on symptoms related to angina*

Table 1 shows how the intervention treatments work for patients presenting with each of the four symptom combinations.

**Table 1:** Efficacy assessment by category of single symptom combination

**Type of symptom combination**	**Four groups of patients categorized using the efficacy assessment tool**			
	**Group 1**	**Group 2**	**Group 3**	**Group 4**
Combination 1	AB1/*N*	A1/*N*	B1/*N*	ab1/*N*
Combination 2	AB2/*N*	A2/*N*	B2/*N*	ab2/*N*
Combination 3	AB3/*N*	A3/*N*	B3/*N*	ab3/*N*
Combination 4	AB4/*N*	A4/*N*	B4/*N*	ab4/*N*

AB1 to AB4 refer to the number of patients requiring treatment for symptom combinations 1 to 4 and for whom both QSYQ and TXL are effective. A1 to A4 refer to the number of patients requiring treatment for symptom combinations 1 to 4 and for whom QSYQ is effective and TXL is ineffective. B1 to B4 refer to the number of patients requiring treatment for symptom combinations 1 to 4 and for whom QSYQ is ineffective and TXL is effective. ab1 to ab4 refer to the number of patients requiring treatment for symptom combinations 1 to 4 and for whom both QSYQ and TXL are ineffective *N* refers to the total number of patients who fall within each symptom combination group, which is planned to be 40.

Table 2 shows how the intervention treatments will work for patients presenting with two or more of the four symptom combinations. All possible symptom combinations are presented in the table. The total number of patients acting as the denominator increases with the number of symptom combinations involved.

**Table 2:** Efficacy assessment by category of multiple symptom combinations

**Multiple symptom combinations**	**Four groups of patients categorized using the efficacy assessment tool**			
	**Group 1**	**Group 2**	**Group 3**	**Group 4**
Combination (1 + 2)	(AB1 + AB2)/2 *N*	(A1 + A2)/2 *N*	(B1 + B2)/2 *N*	(ab1 + ab2)/2 *N*
Combination (1 + 3)	(AB1 + AB3)/2 *N*	(A1 + A3)/2 *N*	(B1 + B3)/2 *N*	(ab1 + ab3)/2 *N*
Combination (1 + 4)	(AB1 + AB4)/2 *N*	(A1 + A4)/2 *N*	(B1 + B4)/2 *N*	(ab1 + ab4)/2 *N*
Combination (2 + 3)	(AB2 + AB3)/2 *N*	(A2 + A3)/2 *N*	(B2 + B3)/2 *N*	(ab2 + ab3)/2 *N*
Combination (2 + 4)	(AB2 + AB4)/2 *N*	(A2 + A4)/2 *N*	(B2 + B4)/2 *N*	(ab2 + ab4)/2 *N*
Combination (3 + 4)	(AB3 + AB4)/2 *N*	(A3 + A4)/2 *N*	(B3 + B4)/2 *N*	(ab3 + ab4)/2 *N*
Combination (1 + 2 + 3)	(AB1 + AB2 + AB3)/3 *N*	(A1 + A2 + A3)/3 *N*	(B1 + B2 + B3)/3 *N*	(ab1 + ab2 + ab3)/3 *N*
Combination (1 + 2 + 4)	(AB1 + AB2 + AB4)/3 *N*	(A1 + A2 + A4)/3 *N*	(B1 + B2 + B4)/3 *N*	(ab1 + ab2 + ab4)/2 *N*
Combination (1 + 3 + 4)	(AB1 + AB3 + AB4)/3 *N*	(A1 + A3 + A4)/3 *N*	(B1 + B3 + B4)/3 *N*	(ab1 + ab3 + ab4)/3 *N*
Combination (2 + 3 + 4)	(AB2 + AB3 + AB4)/3 *N*	(A2 + A3 + A4)/3 *N*	(B2 + B3 + B4)/3 *N*	(ab2 + ab3 + ab4)/3 *N*
Combination (1 + 2 + 3 + 4)	(AB1 + AB2 + AB3 + AB4)/4 *N*	(A1 + A2 + A3 + A4)/4 *N*	(B1 + B2 + B3 + B4)/4 *N*	(ab1 + ab2 + ab3 + ab4)/4 *N*

### Correspondence analysis

Correspondence analysis (CA) is a statistical technique designed to demonstrate or summarize a set of data in two-dimensional graphical form, typically a biplot, which can help in the detection of the structural relationships among the variables contained in the rows and columns of a contingency table. In this study, SPSS version 16.0 software (SPSS Inc, Chicago, IL, USA) will be used to perform CA of the data in Tables 1 and 2. Statistical analysis will follow the steps listed below:

1. Specify the value of dimensions.

2. For both tables, the number of dimensions will be set at two.

3. Production of a CA plot.

The biplot is a matrix of joint plots of the row and column points. In the biplot, types of symptom combinations and different outcome groups are shown as points. The abscissa value is determined by the score of the first dimension of symptom combinations and outcome groups. The ordinate value is the score of the second dimension of symptom combinations and outcome groups.

4. Calculation of “D (A–B)” refers to the relative distance between point A and point B in the biplot. “a1” and “b1” refer to the abscissa values of point A and point B. “a2” and “b2” refer to the ordinate values of point A and point B.

5. The relative distance between points displayed in the CA plot will be summarized in a table. As prespecified, the pair of symptom combinations and outcome types with the nearest relative distance will signify the most significant correlation. The characteristic indications for which the patent drug is most effective will be found upon careful screening.

6. The value of relative distance between two points in the biplot will be calculated using the following formula.

$$D\left(A‒B\right)=\sqrt{{\left(a1\;‒\;b1\right)}^{2}+{\left(a2-b2\right)}^{2}}$$

#### *Safety*

Adverse events (AEs) and adverse drug reactions (ADRs) will be monitored and reported throughout the study. The incidence of AEs and ADRs will be compared between groups using the *χ*^2^ test with the level of significance set at *P* < 0.05.

#### *Drug management*

The central drug administrator will be responsible for drug distribution to and reclamation from each center. Drug administrators at each center will hand out test drugs to the patients and reclaim unused drugs. A drug management log book will be kept.

#### *Data management*

Oracle Clinical Version 4.6 software (Oracle, Redwood Shores, CA, USA) will be used for clinical data management.

## Discussion

TCM syndrome differentiation is the key to understanding the condition or disease and also to choosing the best means of treatment according to traditional Chinese beliefs. It is also held that the efficacy of TCM is most obviously observed in alleviating or improving the characteristic symptoms of patients diagnosed with the corresponding TCM syndrome.

We will conduct a multicenter, randomized, double-blind, double-dummy trial to compare the effectiveness of two Chinese patent drugs, namely, QSYQ and TXL, for angina patients with qi deficiency and blood stasis syndrome.

The study features the grouping of patients with varied treatment needs, the integration of PIO and PRO in efficacy assessment and the use of CA in identifying the most significant correlations between different symptom combinations and treatment outcomes. The ultimate goal of this exploratory study is to establish a methodology for the precise identification of the characteristic indications for which a Chinese patent drug is most effective, and thus differentiation from other drugs used for similar clinical indications in clinical use.

### Strengths and limitations

#### *Strengths*

The following are the potential strengths of our study protocol:

1. Evaluation and interpretation of the therapeutic effects of Chinese patent medicine from the patient’s own perspective

2. Highlighting of individualized evaluation of Chinese patent medicine.

3. Analysis of the curative effects of Chinese patent medicine on single- or multiple-symptom combinations

4. Visualized display of the correspondence between the drug’s efficacy and a specific indication or group of indications

5. Comparison of the therapeutic effects of Chinese patent drugs with other drugs used for similar clinical

#### *Limitations*

The proposed methodology needs to be tested for practicality and feasibility in practice.

#### *Implications*

By comparing the effects of two kinds of Chinese patent medicine on angina symptom improvement, we aim to establish an innovative evaluation methodology that can facilitate the widespread and rational use of Chinese patent drugs by practitioners of TCM and Western medicine.

## Competing interests

The authors declare that they have no competing interests.

## Authors’ contributions

HBC and WM conceived of and designed the study, collected and analyzed the data and wrote the manuscript. JBZ, XL, HXC and CXL collected and analyzed the data and critically revised the manuscript. HCS conceived of and designed the study, obtained financial support and wrote the manuscript. All authors read and approved the final manuscript.
